# Active endothelial and neural regulation of valve biology, health, and disease

**DOI:** 10.3389/fcvm.2026.1768642

**Published:** 2026-05-29

**Authors:** G. Janani, Kenny L. Nguyen, Jonathan T. Butcher

**Affiliations:** Meinig School of Biomedical Engineering, Cornell University, Ithaca, NY, United States

**Keywords:** CAVD, lymphatics, neural cells, physiology, valvular endothelium

## Abstract

Cardiac valves are complex, living, yet passive structures whose function depends on their ability to withstand and respond to dynamic hemodynamic forces through coordinated interactions among specialized cell types and regulatory systems. Traditional models of valve biology emphasize the roles of valve endothelial cells (VECs), valve interstitial cells (VICs), immune populations, and a stratified extracellular matrix in maintaining structural integrity and homeostasis. Recent studies have uncovered underappreciated contributors to valvular physiology, including lymphatic vessels and innervation of nerve fibers that contribute to the interstitial fluid balance, immune cell surveillance, metabolic waste removal, leaflet contractility, and homeostasis. The increasing number of animal studies in the last decades has shed light on the processes of lymphangiogenesis and neural development and their function in cardiac valves. Although developmental and homeostatic functions of these systems are increasingly recognized, their roles in pathological settings such as calcific aortic valve disease (CAVD) remain poorly defined. Disease-associated remodeling may obstruct lymphatic vessels, alter neurofilament organization, and exacerbate inflammatory and fibrotic responses that promote calcification. By integrating these understudied endothelial, lymphatic, and neural components into the broader framework of valve biology, this review highlights critical gaps in current understanding and underscores the potential of these systems as sources of early biomarkers, mechanistic insights, and therapeutic targets for next-generation valve repair and tissue engineering strategies.

## Introduction

The anatomical and functional understanding of the aortic valve and aortic root has evolved substantially since the first detailed descriptions by Leonardo da Vinci, whose studies in the early 16th century recognized the importance of sinus geometry in governing cusp motion (1). Modern structural analyses have confirmed that the valve's thin, trilaminar cusps and three-dimensional architecture form an integrated biomechanical complex that ensures unidirectional flow and minimizes energy loss across the billions of heartbeats over a lifetime (2–4). Microscopic and functional studies demonstrate that valves are not passive flaps but living tissues that respond dynamically to hemodynamic and humoral cues during each cardiac cycle (5–8). Valvular heart diseases are increasing worldwide, with degenerative forms such as calcific aortic valve disease (CAVD) and degenerative mitral valve disease (DMVD) most prevalent in high-income countries, while rheumatic heart disease remains concentrated in low- and middle-income regions. This pattern reflects both demographic trends and differences in healthcare access, with developing countries disproportionately affected by rheumatic etiologies and the global burden of degenerative valve disease rising steadily (9–11). Despite these rising case numbers, disability-adjusted life years attributable to valve disease have declined over the past three decades, which suggest improvements in diagnosis, treatment, and management. Nevertheless, valvular disease remains underdiagnosed in much of the world, and current intervention strategies occur late in the disease course typically after irreversible ventricular damage. Procedures such as the Ross operation demonstrate that living valve autografts can preserve lifespan and maintain native cellular and tissue functions (12, 13). These outcomes support the valve as a self-contained organ with endogenous cellular programs that are critical for long-term function. Currently, no pharmacological therapies have been clinically approved to slow or reverse valve disease. Large trials of atherosclerosis-targeted agents such as statins have failed to treat disease progression, which further highlights that valvular pathology arises from locally unique biological mechanisms rather than systemic atherosclerosis (14). Recent therapies involving Ataciguat, a soluble guanylate cyclase activator, have shown promise in early-phase clinical studies but has not yet received approval (15). Understanding this therapeutic gap requires consideration of broader global landscape of valvular disease, where differing epidemiologic and etiologic patterns predominate across regions.

### Aortic stenosis and mitral regurgitation as dominant valvular pathologies

Left-sided valvular heart disease is more prevalent compared to right-sided disease, due to sustained exposure of systemic pressure and high-velocity flow on the aortic and mitral valves (16–18). This elevated hemodynamic burden imposes substantial mechanical stress on the valve leaflets and drives microscopic injury, endothelial disruption, and maladaptive activation of resident interstitial cells. As these processes accumulate, local matrix remodeling and stiffening further distort the mechanical environment, thereby exacerbating a self-perpetuating feedback loop in which biomechanical strain and structural deterioration reinforce one another, ultimately accelerating the transition from early valve remodeling to stenosis or regurgitation (19, 20). Specifically, aortic valve stenosis (AS) and mitral valve regurgitation (MR) are the two most clinically significant valvular diseases.

Aortic valve stenosis results from progressive calcification and stiffening of the cusps that restrict valve opening. Pathological remodeling typically occurs in the fibrosa layer of the leaflets on the aortic side, in which altered endothelial shear triggers early sub-endothelial injury and inflammation. Over time, these sites transition into macroscopic calcific lesions bridging the leaflet cusps and extending into the sinuses of Valsalva that eventually result in a functional narrowing of the aortic orifice (21). Epidemiologically, the prevalence of AS rises steeply with age as ∼25% of adults ≥65 years of age show leaflet calcification or thickening (22). The development of advanced calcific aortic stenosis is slower with ∼10% in adults >75 years of age demonstrating moderate to severe AS (22–25). Beyond aging, early localization of calcific loci to regions of high mechanical stress suggests a mechanobiological contribution in valvular disease initiation and progression. The influence of the mechanical environment on AS onset is particularly evident in bicuspid aortic valves (BAV), where asymmetric flow and abnormal leaflet geometry impose elevated shear and tensile loads that accelerate calcific remodeling (26). Biomechanical and computational studies confirm that BAV generates abnormal wall shear and higher transvalvular pressure gradients compared to tricuspid valves (27–29). Additionally, sex-differences are evident in the tissue composition of AS with men on average accumulating greater valvular calcium burden, whereas women demonstrate greater fibrotic remodeling (i.e., more collagen and non-mineralized matrix) for the same level of calcification or hemodynamic severity (30). This suggests that female valves may follow a different remodeling trajectory that is fibrosis predominant rather than purely mineralization-driven disease. Collectively, these observations illustrate that AS develops through an intricate interplay between mechanical stress and tissue remodeling that also extends to other forms of valvular diseases such as mitral valve regurgitation. This same paradigm extends to other valvular pathologies such as mitral valve regurgitation.

Mitral valve regurgitation results from structural degeneration of the leaflets and supporting apparatus, which is characterized by disruption of collagen fiber network and accumulation of glycosaminoglycans within the extracellular matrix (ECM) (31). This remodeling leads to disorganization of the connective tissue and impair leaflet coaptation during systole (32). Epidemiological analyses indicate that at least 10% of the general population and approximately 15% of adults > 50 years of age exhibit mitral valve disease, predominantly in the form of MR (33, 34). Despite differences in morphology and hemodynamic consequences, both aortic stenosis and mitral regurgitation share a foundation of maladaptive remodeling driven by chronic mechanical stress and aberrant cellular signaling within the valvular microenvironment. Understanding the native biology of valve tissue and the mechanisms driving such degenerative changes is essential for the development of targeted pharmacological strategies aimed at slowing disease progression or promoting endogenous repair.

Among the resident cell types, valvular interstitial cells (VICs) represent the predominant mesenchymal population within the valve and play a central role in maintaining structural homeostasis. Once considered a largely quiescent fibroblastic cell type, VICs are now recognized for their remarkable plasticity, exhibiting dynamic transitions between fibroblast-, myofibroblast-, and osteoblast-like states in response to biomechanical and biochemical cues (35). Through these phenotypic shifts, VICs orchestrate matrix synthesis, degradation, and mineralization that contributes both to maintenance and maladaptation in valve disease (19, 35). However, growing evidence suggests that other resident cell types are equally integral to valvular health. Pioneering work led by Adrian Chester and his colleagues established endothelial and neural phenotypes in the aortic valve. Through direct neuronal stimulation of isolated porcine cusps, they demonstrated that neuronal-derived nitric oxide (NO) signaling in the aortic valve endothelium actively modulates leaflet stiffness and sustains cusp relaxation (36). More importantly, serotonin induction causes rapid and reversible relaxation in cusp, which suggests a neurotransmitter-like, endothelium-dependent modulation of cusp stiffness and provides the physiological basis for neuro-endothelial crosstalk in valvular functions (6). Additionally, Ohashi et al. previously reported that acetylcholine triggers both transient and sustained hyperpolarization in valve endothelial cells via calcium-dependent activation of potassium channels, which in turn promotes the release of vasoactive mediators such as nitric oxide, endothelin-1, and prostaglandins (37). Together, these insights underscore that valvular homeostasis and disease is not driven by a single cell population, but by coordinated and dysregulated interactions among multiple heterogeneous cell types (5, 38). This review will therefore focus on these less-studied endothelial and neural cell populations and explore their mechanistic contributions to both valve physiology and disease progression, with an eye toward harnessing their properties for future therapeutic interventions.

### The unique phenotype of valvular endothelial cells

**Figure 1:**
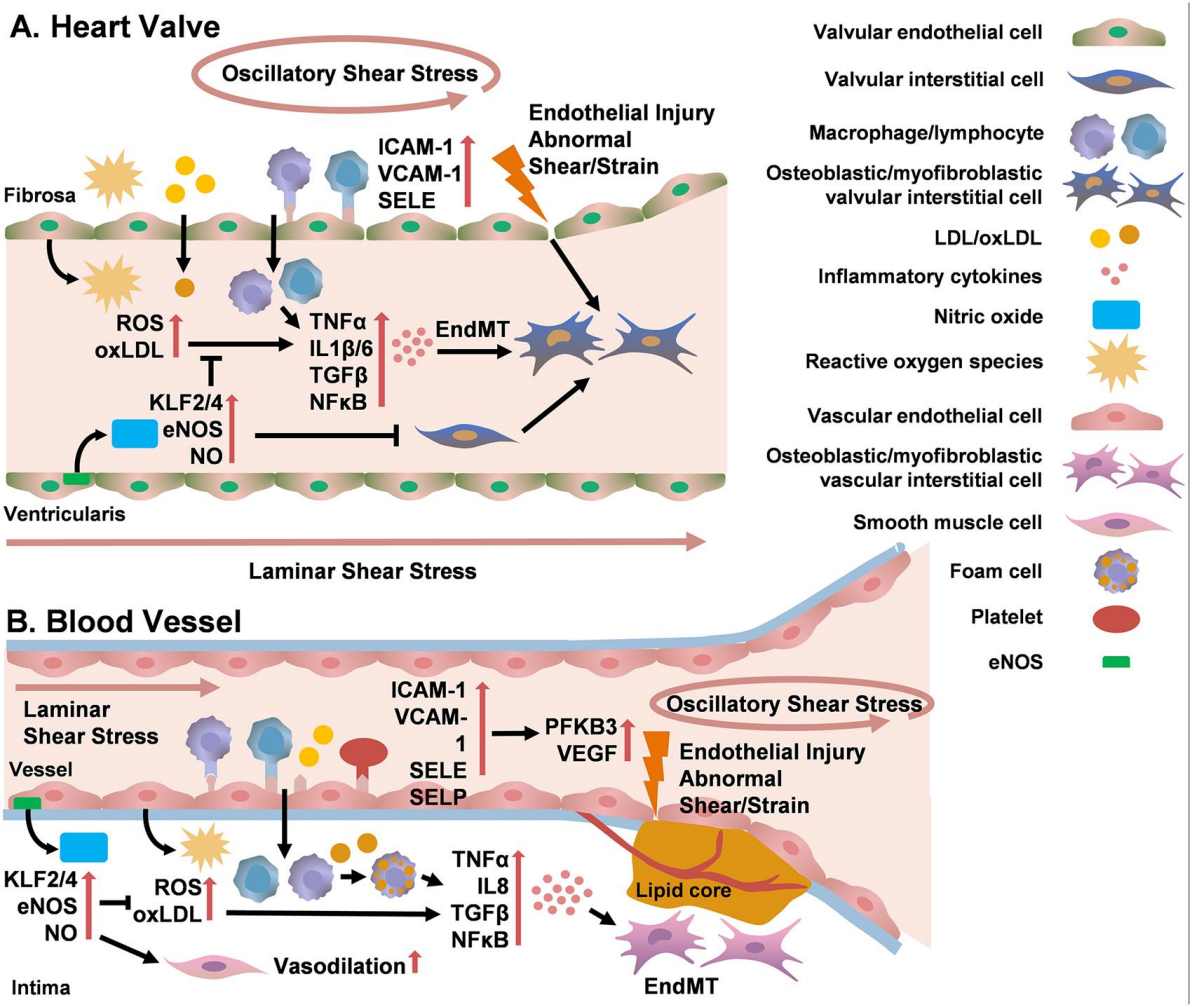
Endothelial cell responses to mechanical and inflammatory cues in the valve and vasculature. **(A)**. Schematic of valvular endothelial cell (VEC) responses to their mechanical microenvironment. On the fibrosa surface, oscillatory shear stress induces a pro-inflammatory phenotype characterized by elevated expression of adhesion molecules (ICAM1, VCAM1, and SELE) and increased production of reactive oxygen species (ROS). External oxidative insults and intrinsic ROS-generating pathways further amplify inflammatory signaling, promoting downstream pathways such as TNF*α*,IL1*β*,TGF*β*,NF*κ*B. These responses shift the VEC–VIC axis toward loss of homeostatic control. In contrast, laminar shear stress on the ventricularis surface enhances protective, anti-calcific, and pro-fibrotic programs that help maintain VIC quiescence and support matrix homeostasis. **(B)**. Vascular endothelial cell responses under disturbed flow. Oscillatory shear stress in arteries similarly upregulates adhesion molecules and is accompanied by heightened SELE expression and enhanced platelet adhesion. This inflammatory state drives metabolic reprogramming, including increased expression of the glycolytic regulator PFKFB3, which cooperates with VEGF signaling to promote angiogenesis in regions adjacent to atherosclerotic plaques. Nitric oxide (NO) production in the vasculature plays a dominant role in modulating vasodilation and vascular tone. Disturbed flow also favors lipid deposition and foam-cell accumulation, further escalating local inflammation in developing plaques.

Endothelial cells constitute a highly specialized interface that governs vascular and tissue homeostasis by integrating mechanical forces, metabolic demands, and paracrine signaling. Unlike the vascular endothelium, valvular endothelial cells (VECs) form a confluent surface monolayer on avascular leaflets and exhibit distinct morphology with overlapping protrusions, fewer tight junctions, and an incomplete basal lamina (39–41). Additionally, VECs exhibit higher proliferative capacity and are uniquely adapted to the side-specific shear and oscillatory flow patterns of the fibrosa and ventricularis surfaces, in contrast to the continuous laminar flow experienced by vascular cells (42, 43). Under fluid flow, VECs align perpendicular to the direction of shear through Rho-kinase signaling, while vascular endothelial cells align parallel to flow through Rho-kinase and PI3-kinase signaling (39, 41, 44). VECs display side-specific mechanosensitive responses: on the ventricularis, exposure to unidirectional, high-shear flow promotes protective transcriptional programs and nitric oxide (NO) production, whereas the fibrosa experiences lower oscillatory shear magnitudes associated with reduced NO and pro-inflammatory, pro-osteogenic signaling (45–48). Inflammatory cytokines such as TNF-α stimulate reactive oxygen species production in VECs, which reduces endothelial nitric oxide synthase (eNOS)/NO signaling and disrupts cell-cell junctions. These same cytokines also drive endothelial activation, characterized by upregulation of ICAM-1, VCAM-1, and E-selectin, promoting leukocyte recruitment and dysfunction (49, 50) (Figure 1A).

Endothelial cells are metabolically specialized to meet distinct functional demands, and early expression profiling indicates that VECs diverge from vascular endothelial cells in ways that are both transcriptionally and functionally meaningful for valve homeostasis and disease (45). In systemic and microvascular endothelium, glycolysis is the principal ATP-producing pathway even under normoxia (51). The preferential reliance on glycolytic pathways enables endothelial cells to generate ATP rapidly to support membrane remodeling and angiogenic sprouting, while simultaneously minimizing reactive oxygen species production. Key glycolytic regulators such as PFKFB3 control tip/stalk behavior during angiogenesis and modulate endothelial proliferation and motility (52) (Figure 1B). Vascular endothelial cells also perform specialized metabolic functions such as insulin transcytosis, facilitating glucose uptake in muscle and adipose tissues, a feature largely irrelevant to the avascular and mechanically specialized environment of the valve (53). While direct metabolic characterizations of VECs are relatively scarce, functional data in human valve endothelial cells demonstrated that under inflammatory stimulation, blocking glycolysis or glucose deprivation shifted VEC energy reliance toward oxidative phosphorylation. Under these conditions, ATP synthase activity was necessary for leukocyte adhesion to VECs via glycoprotein synthesis, suggesting that, like vascular endothelial cells, VECs may retain metabolic flexibility, with mitochondrial oxidative metabolism becoming more important under stress (54). These distinctive properties enable VECs to sense complex hemodynamic cues and modulate interstitial cell behavior and matrix organization. In *ex vivo* cusp preparations, VEC-derived mediators such as NO, serotonin, and endothelin dynamically modulate cusp stiffness and contractility which preserve the leaflet's physiological compliance under cyclic loading. Loss or dysfunction of the VEC layer abolishes these modulatory effects further supporting endothelial contribution to tissue tone (6).

Tensional homeostasis is governed by coordinated mechanical activity between VECs and VICs. *In vitro* work using 3D hydrogels with tunable matrix demonstrated that VICs actively regulate the internal stress state of the leaflet by modulating their contractility and ECM in response to substrate stiffness. On compliant matrices mimicking physiological valve environment, VICs adopt a quiescent, fibroblast-like phenotype characterized by low contractile tone, preserved matrix signaling, and minimal osteogenic signaling. However, when cultured within lowered initial stiffness matrices, VICs transition towards an osteogenic phenotype characterized by elevated contractility, increased alkaline phosphatase activity, and higher RUNX2 and osteocalcin expression (55–57). Complimentary studies define that VIC contractility is essential for preserving organized collagen architecture by aligning collagen fibers along tensile forces to sustain internal mechanical equilibrium. Osteogenic treatment disrupted VIC contractility and led to disorganization of the collagen matrix with increased RhoA signaling, *α*SMA, TGF-*β*, and RUNX2 (58). VECs serve as a critical modulator in the valve by acting as a biomechanical buffer that titrates VIC-generated stress under physiological conditions, but under inflammation or pathological strain, VEC regulatory capacity is diminished and augments VIC activation (59). *In vitro* and *ex vivo* work shows that endothelium-derived NO signaling modulates VIC matrix production and suppresses osteogenic differentiation (60, 61). Endothelial NO signaling contributes to valvular homeostasis by modulating NOTCH1 activity and restraining interstitial cell activation (62). In co-culture models, NO produced by VECs limits osteogenic differentiation of VICs, whereas inhibition of NO signaling accelerates calcification, an effect reversed by restoring NOTCH1 expression in VICs (61). Conversely, endothelial injury or disturbed shear promotes paracrine cues that permit VIC myofibroblastic activation and osteogenic conversion. *In vivo* studies show endothelial nitric oxide synthase (eNOS) deficiency increases the risk of bicuspid aortic valve in mice with ∼30% of eNOS ^−/−^ developing bicuspid valves (63).

Cyclic mechanical strain and inflammatory cues induce cytoskeletal and focal adhesion remodeling in endothelial cells and trigger partial or full endothelial-to-mesenchymal transition (EndMT). This process generates a population of activated fibroblastic-like cells in the interstitium, which promotes ECM remodeling and the formation of calcific lesions. Experimental models demonstrate that cyclic stretch at different loading regimes induces EndMT and promotes two distinct mesenchymal phenotypes, where lower strain amplitudes preferentially engage TGF−*β*1 signaling and higher strain amplitudes are predominated by *β*−catenin/Wnt signaling (64). Cadherin-11 (Cad-11) is a mesenchymal cadherin that mediates calcium-dependent cell-cell adhesion and regulates cushion compaction and mesenchymal organization during cardiac valve morphogenesis (65–67). In the mature mice valves, Cad-11 expression becomes largely restricted to the endothelial layer and contributes to adhesive signaling pathways that maintain tensional homeostasis by modulating intercellular force transmission between VECs and underlying VICs. Cad-11 overexpression amplifies RhoA-GTP signaling, increases Sox9, drives ECM remodeling, and calcification (68). Conversely, Cad-11-null valves exhibit reduced cell migration, impaired contractility, and preserved resistance to calcification despite progressing stenosis. Transfection of Cad-11 depleted cells with constitutively active RhoA restored contractile and migratory features (67). Under pathological conditions, Cad-11 is observed to predominantly regulate RUNX2 nuclear localization through Rac1 and Rac1-GEF signaling. Rac1-GEF inhibition via NSC23766 significantly reduces calcification in *ex vivo* porcine aortic valve in osteogenic media (69). Regulatory transcription factors such as KLF2 and KLF4 integrate shear-dependent protective signals; endothelial deletion of either factor in murine valves reproduces hallmarks of myxomatous degeneration, which highlights the importance of hemodynamic-responsive gene programs in maintaining leaflet structure and suppressing pathological (70).

Although VECs and VICs share overlapping signaling pathways, the regulation and functional outcomes often diverge between the two cell types. NOTCH1 signaling in VECs plays a critical role in both valve development and adult homeostasis. Mutations in NOTCH1 are causally linked to CAVD, and VEC-specific NOTCH1–RBPJ activity regulates inflammatory mediators, apoptosis, and proliferation of adjacent VICs. In this context, NOTCH1 restricts osteogenic cues, including BMP2 expression in VICs, thereby exerting anti-calcific effects (71). NOTCH1 signaling in human valvular endothelial cells directly regulates Matrix Gla Protein (MGP) and other calcification-inhibitory gene networks, thereby integrating endothelial mechanotransduction with the suppression of pro-calcific signaling (72). Similarly, VECs serve as a critical paracrine node through endothelial-derived TGF-*β*1 signaling, which preserves SOX9 nuclear localization in neighboring VICs and attenuates osteogenic differentiation and calcific nodule formation (73). By contrast, VICs are the primary effector population for BMP- and Wnt-driven osteogenic programs as exposure of human VICs to BMP-2 activates SMAD1 and ERK signaling and rapidly up-regulates RUNX2 and osteopontin (74).

### Lymphatic connections to VEC

**Figure 2:**
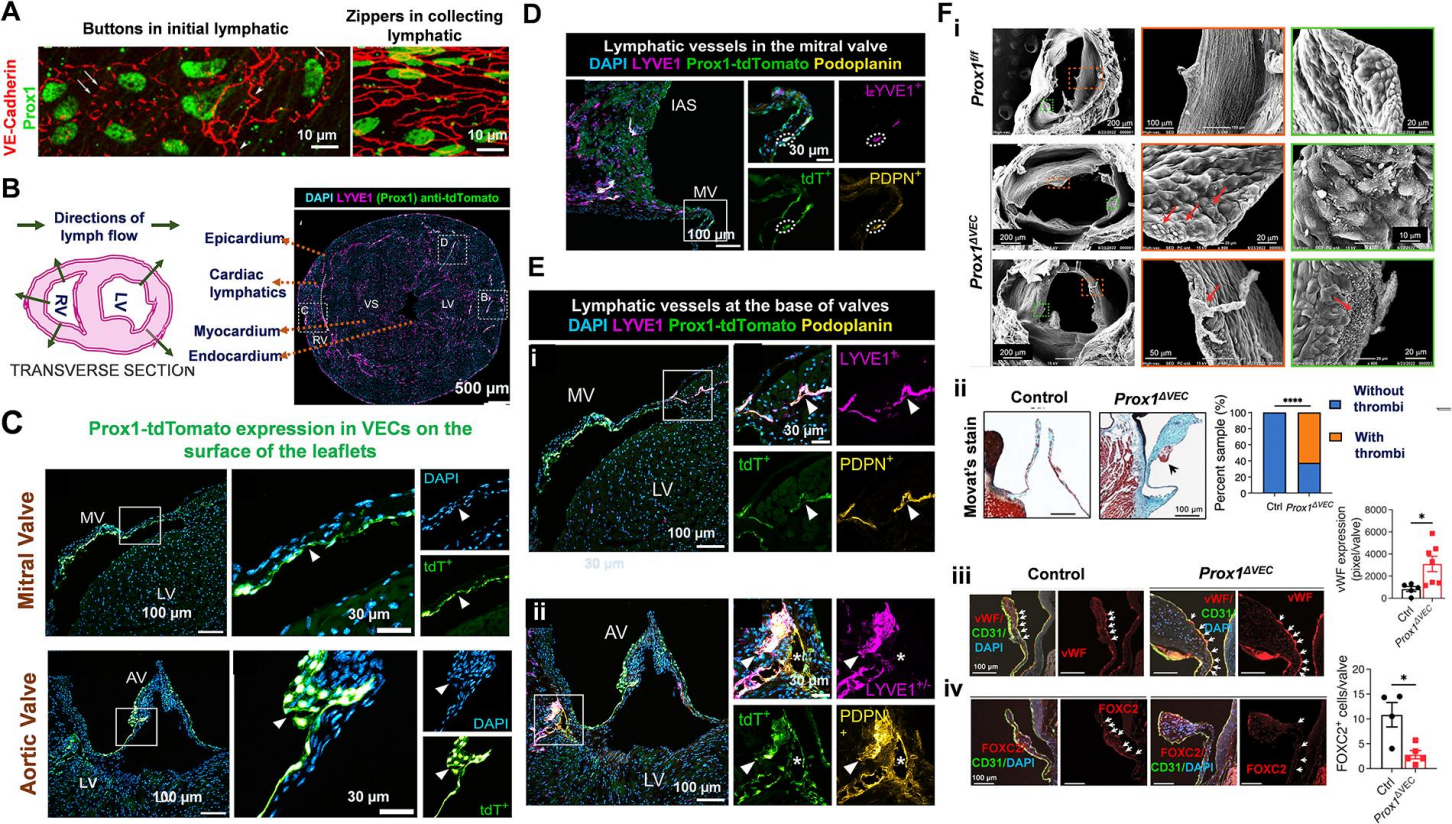
Lymphatic function in cardiac valves **(A)**. Confocal images of Prox1 (green)-positive lymphatic networks showing VE-cadherin (red)-positive “button-like” and “zipper” junctions in initial and collecting lymphatic vessels, respectively. Adapted from “Buttons in endothelium of initial lymphatics” by Baluk *et al*., licensed under CC BY-NC-SA 4.0. **(B)** Cardiac contractions drive lymphatic drainage. RV - Right ventricle, LV - Left Ventricle. Tile scan of transverse view of Prox1-tdTomatoTg/+ mice stained for LYVE1 and Prox1 showing ventricular lymphatics enriched in epicardial surface. Adapted from “Ventricular lymphatics are enriched on the epicardial surface, subepicardial region, and right ventricular septum” by Ware *et al.*, licensed under CC BY-NC 4.0. **(C)** Prox1-tdT (green) expression on the surface of mitral and aortic valve primarily by VECs and not VICs. Adapted from “Supplemental Figure S13. Prox1-tdTomato expression on the surface of the valves” by Ware *et al.*, licensed under CC BY-NC 4.0. **(D)** Presence of lymphatic vessels in the mitral valve stained for LYVE1 (magenta), PDPN (yellow), and Prox1-tdT (green). Adapted from “Lymphatic vessels in the left atrium, interatrial septum, mitral valve, and near the tricuspid valve and atrioventricular node” by Ware *et al.*, licensed under CC BY-NC 4.0. **(E)** Presence of lymphatic vessels at the base of (i) mitral valve and (ii) aortic valve stained for LYVE1 (magenta), PDPN (yellow), and Prox1-tdT (green). Adapted from “Supplemental Figure S12. Lymphatic vessels near the mitral, aortic and pulmonary valves” by Ware *et al.*, licensed under CC BY-NC 4.0. **(F)** PROX1 deletion in VECs results in valve thickening and thrombus formation. (i) Representative scanning electron microscope (SEM) images of the tri-cuspid aortic leaflets showing Prox1ΔVEC mice have thicker aortic valves, disrupted endothelial layer (arrows in the middle row) with infiltration of platelet-like cells (arrows in the bottom row), (ii) Representative Movat Pentachrome-stained images demonstrating thrombus structure in the aortic valve of Prox1ΔVEC mice, (iii) vWF (red) expression was increased in the downstream side of VECs of Prox1ΔVEC mice (arrows) and (iv) FOXC2 (red) expression was reduced in Prox1ΔVEC mice. Reproduced from “Loss of PROX1 (Prospero-related homeobox transcription factor 1) from aortic valvular endothelial cells (VECs) results in valve thickening, damaged endothelium, thrombus formation, and downregulation of FOXC2 (Forkhead box C2 transcription factor) in VECs” by Ho *et al.* licensed under CC BY-NC-ND 4.0.

A branched lymphatic vessel networks comprising capillaries and lymph nodes, are lined with a thin layer of lymphatic endothelial cells (LECs) with discontinuous “button-like” cell junctions in initial stage and “zippers” in collecting lymphatic vessels, that act as “flap valves” in relation to the changes in interstitial pressure (75) (Figure 2A). Lymphatic system plays integral role in the circulatory system regulating interstitial fluid homeostasis, immune cell trafficking, lipid transport, and distributing nutrients (76, 77). LECs exhibit a distinct expression profile to blood vessels by presenting the vascular endothelial growth factor receptor 3 (VEGFR-3), neuorpilin-2, transmembrane *O-*glycoprotein podoplanin (known as PDPN, T1*α*, gp38, and E11 antigen), and lymphatic vessel endothelial hyaluronan receptor-1 (LYVE1) (77). These lymphatic vessels develop immediately after cardiovascular system at 6–7 embryonic weeks in humans, wherein the endothelial subpopulation in the lateral anterior cardinal veins expressing transcription factors, prospero homeobox-1 (Prox1) sprout to form primitive lymphatic sacs (78). After extensive investigation into lymphatic development over the past decade, recent lineage-tracing studies have highlighted a notable diversity of organ-specific LEC population. More specifically, cardiac lymphatic vessel was shown to be descended from a heterogeneous cell population comprising both venous sprouting and Tie2-lineage^−^ nonvenous LEC progenitors (79). Traditionally, heart valves (atrioventricular and semilunar valves) were thought to be non-vascularized (80), and the blood vessels were found to appear in response to the inflammation (81, 82). The degree or extension of blood vessels in native valves explain the pathogenesis of valvular health, indeed the presence of blood vessels in the explants of engineered valve is crucial during repair and remodeling (83). The pluripotent differentiation capacity of valve endothelium (VECs) generates new blood vessels during rheumatic heart valve disease, describing its ability to generate lymphatic vessels (84–86). Recently, ventricular lymphatic system is largely studied, confirming its presence particularly along the epicardial and subepicardial surface of the left ventricle, as well as within endocardium of right ventricle (87, 88). The heart contractions during diastole propel the lymph flow from subendocardial to myocardial, and systolic contractions drive lymph towards subepicaridal lymphatics from myocardium (87) (Figure 2B). Cardiac lymphatics is important in maintaining heart homeostasis, wherein lymphatic obstruction induced myocardial lymphedema (89) and selective lymphangiogenic therapy using VEGFR3-CC152S microparticles limited cardiac dysfunction, fibrosis and accelerated inflammation (90, 91). Lymphangiogenesis is also associated with chronic inflammation, wherein previous research showed that blocking VEGFR-3 targeted lymphatic vessel activation and CCL21 production in the allograft, providing a novel immunomodulatory mode of therapy in cardiac transplants in a mice model (85).

Highly metabolically active heart valves define the way valve endothelial, interstitial cells and immune cells generate and utilize energy to support their physiological functions including homeostasis, extensive ECM remodeling and opening cycle (92). The hyperglycolytic metabolic shift was noticed in aortic stenotic conditions, however a research using glucose analog ^18^F-fluorodeoxyglucose showed that the metabolism is not correlated with macrophages (93). This raises the question about which other cells can contribute to this metabolism other than macrophages and valve cells (94). Visconti *et al*. (96) demonstrated that bone marrow derived hematopoietic stem cells (Lin^−^, c-kit^+^, Sca-1^+^, CD34^−^) contribute to the adult VICs in addition to the embryonic source cells (95, 96). Hulin *et al*. (98) showed the heterogeneity of myeloid cells and macrophage subpopulations present in heart valves. Especially, nonmyeloid dendritic cells (CD11b− and CD11c+), myeloid macrophages expressing F4/80, CD206+ and F4/80, MHCII increased in number after birth supporting physiological ECM remodeling (97). They also observed minor immune cell population present in the healthy leaflets, such as lymphocytes and neutrophils which remain to be characterized (97). Research studies noticed the positive expression of lymphatic transcription factors, PROX1 and FOXC2 in a subset of VECs located on the fibrosa side of leaflets (98, 99) (Figure 2C). A detailed study on distribution of lymphatic vessels using Prox1-tdTomato lymphatic reporter mouse model by Ware *et al*. (80) showed LYVE1+/Prox1+/PDPN + lymphatic vessels within the mitral valve, and these LYVE1 signal were mainly originated from resident macrophages (80) (Figure 2D**)**. Lymphatic vessels positive for both LYVE1+ and LYVE1- Prox1+/PDPN+ lymphatic vessels were present at the base of the valves, however the VECs in the leaflets were positive only for Prox1 in aligned with the earlier reports (Figure 2E).These findings necessitated the significance of minimal two lymphatic specific markers to affirm lymphatic vessels in heart valves. Ho *et al*. (98) showed that conditional VEC-specific *Prox1* deletion in mice resulted in an enlarged and stenotic aortic leaflets with decreased *FOXC2* and increased PDGF-*β* expression. PROX1 preserves ECM homeostasis by inhibiting PDGF-*β* signaling via FOXC2 and limits myxomatous degeneration in human aortic and mitral valves (Figure 2F). In pathological conditions, especially in human heart valve infective endocarditis, inflammation-associated lymphatic vasculature is markedly increased with that observed in degenerative valves (100, 101). Recent studies have further expanded this finding by identifying a distinct VEGFR3+ lymphatic valve endothelial cell population located on the atrial side of the mitral valve (102, 103). These cells appear to contribute to lymphatic outgrowth in the worsened pathological settings such as human rheumatic heart disease. Altogether, it is conceivable that the lymphatics and angiogenesis plays major role in valve development, homeostasis, and disease progression. Unraveling the interplay in lymphangiogenesis will furnish a better understanding of its importance in valve disease and aid in finding the potential therapeutic targets.

### Neural activity in valves – an old feature

**Figure 3:**
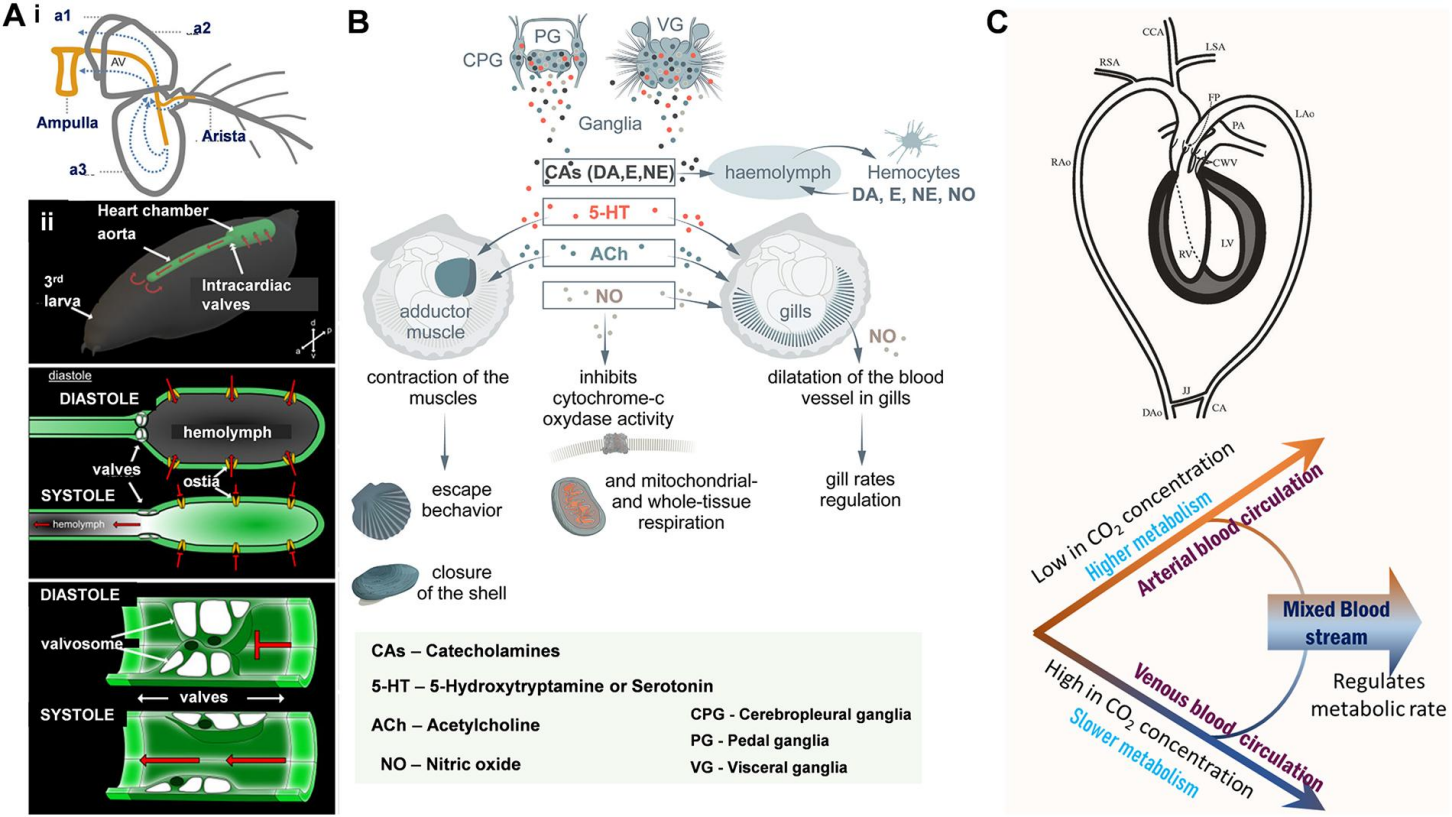
Evolutionary emergence of nerve fibers in cardiac valves **(A)**. (i) Frontal view of Drosophila antenna and ampula showing the hemolymph flow (in blue). Reproduced from “Schematic diagram of the Drosophila antenna and frontal pulsatile organ (FPO). (A) Frontal view of antenna and ampulla, showing the flow of hemolymph (blue arrows)” by Alan R. Kay, Daniel F. Eberl, and Jing W. Wang, licensed under CC BY. (ii) Open Drosophila circulatory system wherein a pair of ostia cells facilitates the entry of hemolymph during diastole and is pumped through aorta upon systole. Adapted from “Malformations of cardiac valves in Drosopila. (A) Open circulatory system in the Drosophila larva” by Christian Meyer and Achim Paululat, licensed under CC BY. **(B)** Role of neuroendocrine signal under hypoxic response in marine bivalve mollusks regulating muscle contraction and dilation of blood vessels in gills. Adapted from “Diagram summarizing available data on the hypoxia stress control and the role of neuroendocrine regulation in the hypoxic response in bivalves. The involvement of CAs, 5-HT, ACh, and NO under hypoxic conditions is shown” by Elena Kotsyuba and Vyacheslav Dyachuk, licensed under CC BY 4.0. **(C)** Crocodilian central circulation system showing cog-wheel valve (CWV) in subpulmonary conus Adapted from “The crocodilian central circulation, ventral view” by Douglas A. Syme, Kurt Gamperl, and David R. Jones, licensed under CC BY 4.0.

While mechanobiological mechanism remain crucial and well-characterized in governing heart valve structure and function, neural activity has emerged as an additional mode of active control and previously underappreciated. Neurosignaling in heart valves may indicate that valve innervation provide additional support in modulating valvular tone, cellular contractility, and extracellular matrix dynamics apart from the hemodynamic forces elicited by rhythmic cardiac contractions (104, 105). Evolutionarily, the emergence of nerve fibers within valves appears to have a conserved mechanism to enhance the precision and adaptability of biomechanical regulation of leaflet functions. The persistence of nerve fibers across species suggests that they also contribute to optimal flow dynamics and neurocardiac feedback is prerequisite for maintaining valve homeostasis. In insects, the neurally activated valve-like “ostia” majorly facilitated bidirectional hemolymph flow opening ostia and filling ampulla, supported by auxillary contractions modulated by neuropeptides and neurotransmitters especially in *Drosophila melanogaster* (106–108) (Figure 3A). The neuroendocrine signaling system in marine bivalve mollusks play essential roles in regulating physiological, biochemical, molecular defenses, and hypoxia stress response to conserve metabolic resources (109–111) (Figure 3B). *Nautilus pompilius L.* cephalopoda demonstrated distribution of neuropeptides related to tachykinins (TKs), vasoactive intestinal polypeptide (VIP) and FMRFamide in the heart, especially FMRFamide immunoreactivity was observed in semilunar atrioventricular valves (112). Surprisingly, the receptor for TKs is located on eNOS positive endothelial cells, and these neuropeptides indirectly regulated cardiac output (112). Similarly, alligators and crocodilians possess a well-developed cog-wheel valves (CWV) located in the subpulmonary conus outside the right ventricle (113). This neutrally activated valve consists of connective tissue nodules surrounded by the cardiac muscle, and contraction of this valve diverts blood flow to retain oxygen, and support relative resistance to stay underwater a long time and support shunting mechanism (113, 114) (Figure 3C). These “evolutionary” elements may confer conserved functions in higher order mammals that are as yet unknown. The functional significance of nerve structures in the heart valve play crucial role in maintenance of its physiological structure and function in response to hemodynamic and mechanical conditions.

**Figure 4:**
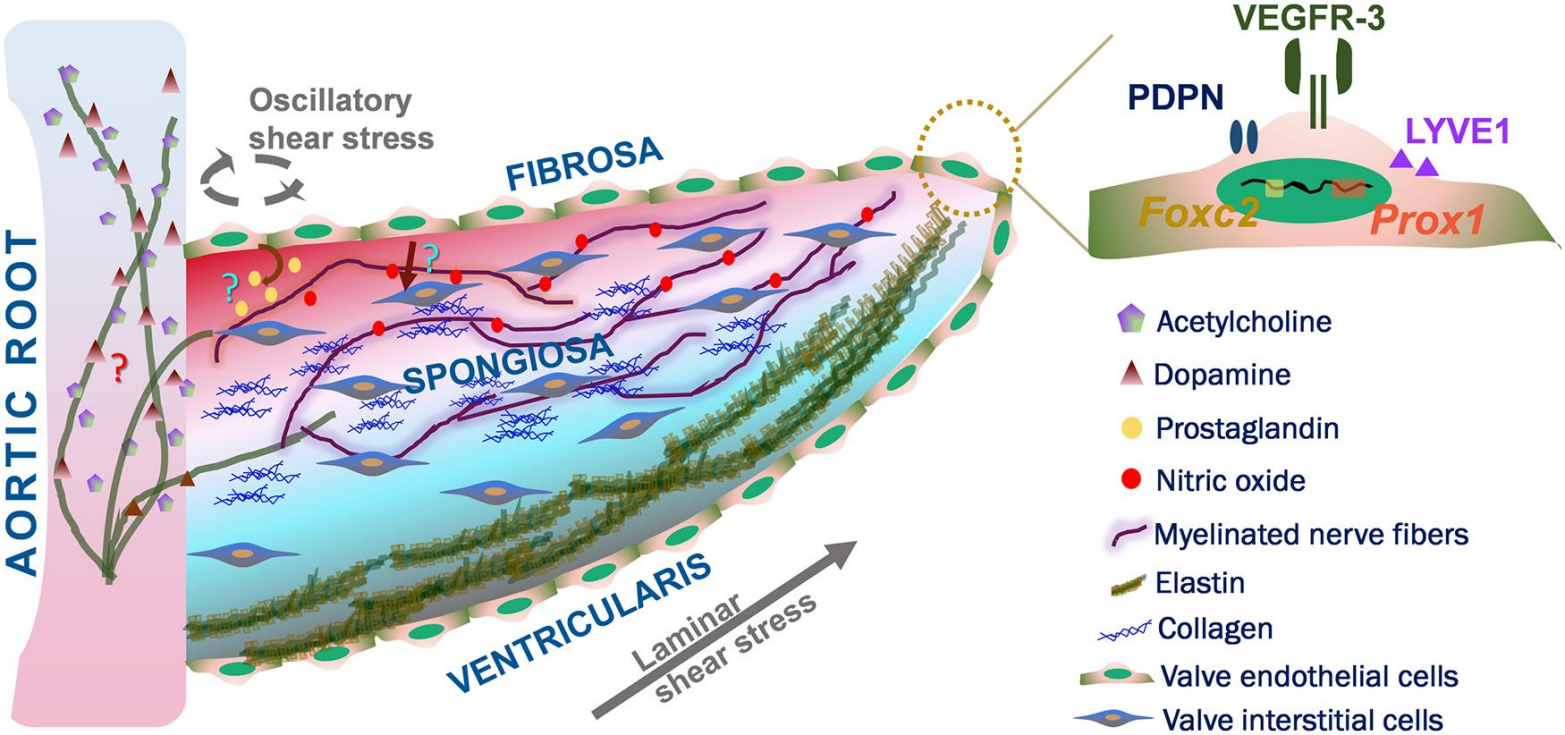
Lymphatic and neural characteristics of aortic valve leaflets. Schematic illustrating lymphatic markers and neural filaments in aortic valve leaflets. Valve endothelial cells (VECs) express lymphatic-associated markers: VEGFR-3, LYVE1, PDPN and transcription factors: *Foxc2* and *Prox1*. VECs also release prostaglandins to the cusp tissue, though its valve-specific function remains unclear. Neurotransmitters showed regional specific expression: the aortic root showed presence of acetylcholine- and dopamine-associated fibers, whereas leaflets demonstrate and nitric oxide-associated myelinated nerve fibers. The functions relevance of prostaglandin and nitric oxide in the leaflets is not yet defined.

Recent evidences indicated that the mitral and tricuspid valves possess intrinsic contractive properties in addition to the classical valvular motion attributed by the coordinated contraction and relaxation activity of the myocardium, annulus, and papillary muscles (115, 116). Early foundations studies identified a rich innervation of primary sensory and autonomic neural components within the valve cusp tissue of dogs and pigs, suggesting unrecognized neuroregulatory mechanism governing valve function (117, 118). Anatomical studies by histochemistry and electron microscopy reveals the first evidence of nerve fibers originating from adventitial wall (119) in human aortic valves (AoV) and arterial valves (120–122), to assist in the development, maturation and maintaining valve homeostasis (36). The sympathetic and parasympathetic responses aids in contraction, especially nitric-oxide containing nerves mediate relaxation of valve cusps (36). Early investigations in 1960's revealed that translucent mitral valve leaflet contains elastic fibers, nonmyelinated nerve fibers, and blood vessels extending toward the free margin of the valve (117, 123). Comparatively, density of nerve terminal arborizations growing from myelinated nerve fibers were dominant in AoV extending over the proximal and medial portions of leaflets, whereas arterial valves possess nerve fibers in ventricular layer. Nerve terminals elicit reflex responses upon prostaglandin stimulation released by AoV endothelial cells (124, 125). *In vitro* experiments with dog mitral valve leaflets showed active contraction and tension development upon electrical stimulation, which was enhanced upon norepinephrine and attenuated by acetylcholine (117). A heterogeneous population of nerve fibers and terminals with distinct innervation patterns were identified in heart valve leaflets within 30–80 nm of rodent interstitial cells (126) and∼300 nm of porcine SMCs (104, 118, 127). Chester et al. (37) characterized the neuronal supply across multiple aortic root regions, including annulus, sinus, sinotubular junction, aortic root and cusp tissues. Neurofilament-positive nerve fibers in aortic root structures were associated with tyrosine hydroxylase and choline acetyl transferase, and neuronal nitric oxide synthase positive nerve fibers in cusps were located in close proximity to endothelial surface (36). Sinus, sinotubular junction, and annulus dissected from porcine aortic roots supported frequency dependent sympathetic and parasympathetic contractions in response to electrical field stimulation, whereas the neuronal nitric oxide synthase positive nerve fibers in the cusp tissue exert a nitric-oxide mediate neurogenic dilator tone supporting the valve cusp relaxation (36, 128). These heterogeneous network of nerve fibers along the aortic root to leaflets are likely responsible for valve opening-closing cycle and stiffness modulation in response to hemodynamics (38). In line with this hypothesis, remote stimulation of the vagal nerve fibers within the sheep mitral valve has been shown to modulate MV leaflet stiffening (129). To the best of our knowledge, the role of valvular neural signaling in regulating homeostasis, inflammation and valvular pathology remain largely unknown and require substantial investigation. Significant knowledge gaps remain regarding the various neurotransmitters present around the valvular leaflets including cusps, aortic root, sinus wall, and leaflets (Figure 4). Human valve-based research providing direct functional evidence linking neural population to valve disease progression remain limited. This necessitates a critical need for further investigation into both lymphangiogenesis and neural regulatory networks within human cardiac valve tissues. Expanding our understanding on these biomolecular mechanisms and multicellular interactions could provide significant insights into their functions in valve health, pathology, and disease progression. Addressing these setbacks will motivate the researchers to investigate the potential therapeutic avenues of neuronal control in cardiovascular research.

### Endothelium and nerves in living valve replacements

Severe aortic valve disease is treated primarily through prosthetic aortic valve replacement (AVR), current gold standard approach, however this intervention is accompanied by significant patient related challenges. Prosthetic valves are of 3 different categories, (i) Surgical mechanical aortic valves constructed from different synthetic material offering a long lifespan of 25–30 years, (ii) Surgical biological aortic valves derived from xenogenic or allogenic tissues lacks durability, and (iii) transcatheter aortic valves employed in less invasive approaches (130). Despite their widespread clinical applications, these prosthetic valves lack bioactivity and are prone to infection, thrombosis and structural valve deterioration. The Ross surgical procedure, an autograft utilizes pulmonary root as a conduit (131) for aortic valve replacement uniquely preserve endothelial and interstitial cell population in contrast to prosthetic replacements. Ross technique maintained functional collagen architecture after 6 years and supported long-term outcomes in young and middle-aged adults (13). Post-implantation, endothelial and interstitial cells in pulmonary autograft undergoes adaptive ECM remodeling to withstand mechanical stress and mimic the native aortic root anatomy, wherein increased endothelial cell population start to strongly express CD31 and Ephrin B2 (arterial endothelial cell marker) (132). However, there is no study validating the presence of nerve terminals retained in Ross procedure autographs (13). The Ross procedure is technically complex and requires open heart surgery and extended cardiac bypass, therefore not suitable for elderly patients (131). Ross is less successful in younger patients due to the dilation of valves rather than valve growth and remodeling.

To overcome these challenges, the development of lab grown heart valve substitutes either with or without living cells that actively adapt to the intrinsic mechanical stress and remodel over time represents a promising solution (133). In this line, the readily available acellular tissue engineered heart valves (TEHV) utilizing decellularized and synthetic matrices is increasing over the years (134). Physiological-like CAD design implicated in second generation TEHV with optimized strain distribution profiles in the leaflets overcomes the limitation of tissue compaction and promotes functional remodeling (135). To achieve proper homeostasis and remodeling, homing of progenitor cells and immune cells from bone marrow and blood is crucial, and the recruited bone marrow derived progenitors showed differentiation to valvular interstitial cell population (134, 136). The immune cells infiltrated towards TEHV implants polarized to inflammatory M1, remodeling M2 phenotypes (137), that activated the recruitment of *α*-SMA positive smooth muscle cells (135, 138). Recently, acellular scaffolds implanted in the pulmonary position supported maturation and active valvular remodeling with appearance of neurogenic tissue development at 3 weeks expressing neurotransmitters and post 12 weeks nerve bundles expressed vasointestinal peptide, tyrosine hydroxylase, neruopeptide Y and neurofilament protein to reduce inflammatory response (139). This is the first tissue engineered valve reporting the presence of nerves during the tissue remodeling phase. Despite advancements in structure design such as incorporating sinuses, the sinotubular junction, and cusp-wall junction, current TEHV still lack integration of the biological mechanism that regulate the leaflet contractile functions, particularly those mediated by valve cells and nerve fibers. These grafts sometimes dilate due to lack of mechanical stiffness, which can be overcome by pre-seeding the scaffold with endothelial cells that can accelerate the regeneration process (140). Endothelial cell pre-seeding on TEHV promoted anti-thrombogenic surface with minimal platelet adhesion, recellularization, reduced inflammation and calcification (141, 142). On the other hand, the recruited M2 macrophages have been reported to undergo macrophage to myofibroblast transition expressing *α*-SMA (143), and endothelialization process of hinge region was completed by 8 weeks. Micropatterned microfluidic channels functionalized with Jag1 and Dll4 ligands on substrates spatially controlled endothelial sprouting (144). The challenges of achieving functional endothelium and nerve fibers in TEHV are multifactorial. First, the complex process of lymphangiogenesis in valve leaflets is technically unclear, requiring additional validation of its significance in valve pathogenesis. Furthermore, lymphangiogenesis can promote faster remodeling of implants overcoming the thickening and inflammation in the conduit. Finally, the long-term role of nerve fibers and endothelium in the TEHV have not yet been established.

### Transcriptomic landscape of diverse cellular communities

Transcriptomic profiling has transformed valve biology by revealing the complex cellular heterogeneity and transitional cell states that underpin both healthy valve function and the pathological transition toward calcification or stenosis. Initial investigations utilized bulk RNA-sequencing (RNA-seq) to establish a foundational understanding of global transcriptional signatures defining disease states. Houessou et al. conducted a large-scale bulk RNA-seq study of human cohorts, comprising over 400 patients, identifying co-expression of gene modules that link lipid metabolism, inflammation, and immune infiltration to disease progression (145). Schlotter et al. conducted a multi-omic approach combining proteomics and bulk RNA-seq on human stenotic aortic valves to establish a regulatory network in CAVD (146). Recent advancements in single-cell RNA sequencing (scRNA-seq) technologies allowed for the opportunity to characterize valve heterogeneity. The first transcriptome atlas of human aortic valves using scRNA-seq identified 14 distinct cell clusters; notably, the identification of specialized VEC and VIC subtypes and their associated transitional states emphasized the role of EndMT as a critical driver of thickening in calcified aortic valve leaflets (147). Villa-Roel et al. investigated the side-specific transcriptomic profiles of human aortic valves using scRNA-seq to further elucidate the distinct hemodynamic environments of the fibrosa and ventricularis (47). To better define the baseline cellular states from which these pathologies emerge, Shu et al. established a transcriptome atlas of healthy human valves. This work elucidated the homeostatic roles of various VEC and VIC subtypes in both semilunar and atrioventricular valves (148). Additionally, other scRNA-seq studies on porcine aortic VICs showed nitric oxide signaling maintains VIC quiescence by modulating integrin-related transcriptional programs (ITGA8, VCL) (149). Beyond the remodeling of structural cell types, scRNA-seq has further elucidated the inflammatory landscape of CAVD by uncovering T-cell heterogeneity in human calcified leaflets (150). While current atlases mapped the structural and immune landscape of the valve, there is a lacking of work that characterizes the transcriptomic signatures of valvular innervation.

## Summary and future directions

Valve biology is shaped by a diverse cellular community, yet many non-interstitial populations remain underappreciated despite their critical roles in homeostasis and disease. VECs and neural elements are central to the regulation of mechanical signaling, cellular crosstalk, and tissue remodeling, but have received far less research attention compared with VICs. This gap limits our ability to develop therapies that selectively target pathological processes, as interventions directed solely at VICs risk unintended effects on neighboring VECs or neural components. Several studies have shown bidirectional VEC-VIC crosstalk, where VICs are not passive recipients of endothelial signals; rather they can reprogram VEC behavior through secreted factors that influence endothelial activation and matrix-related pathways (151, 152). This reciprocal signaling loop is central to maintaining, or disrupting, valvular homeostasis.

Emerging evidence highlights that biological outcomes in the valve are dictated not only by individual cell types but also by the spatial context and interactions among neighboring populations. Mesoscale cellular communities, where endothelial, interstitial, neural, and potentially lymphatic cells interact, play a decisive role in remodeling mechanisms and disease progression. Interestingly, lymphatic vessels present in both atrioventricular and semilunar valves, particularly along the ventricularis and subendothelial regions contribute to fluid homeostasis, immune surveillance, and inflammatory resolution. Further, studies have demonstrated the autonomic innervation of both sympathetic and parasympathetic nerve fibers in the leaflets have been implicated in modulating the valve contractility. Altogether, the present review suggests that integrated mechanobiological, lymphatic, immunological and neuroregulatory mechanisms maintain valve homeostasis, significant gaps persist regarding how lymphatic and neural pathways regulate valvular pathophysiology. Recapitulating these multi signaling networks in tissue engineered heart valves (TEHV) post implantation facilitate test interventions, could yield functional aortic valve replacements capable of mechanosensing, remodeling and long-term functions. Recent advances in TEHVs demonstrate that anatomically precise, acellular synthetic scaffolds can undergo rapid *in situ* morphogenesis involving endothelial, interstitial, neural, and extracellular matrix components, as shown by Yacoub and colleagues (139). These findings highlight the promise of TEHVs as next-generation valve prosthetics and emphasize the need for future designs that optimize function at implantation and better coordinate early hemodynamic remodeling. Incorporating insights from underappreciated cell types will be crucial to guide early hemodynamic adaptation and long-term valve performance. Technologies such as spatial transcriptomics, single-cell transcriptomic profiling, high-resolution imaging, and functional molecular perturbation studies now provide unprecedented opportunities to map these interactions, quantify cell-type-specific contributions, and identify previously unrecognized regulatory networks. It is likely that the contributions of these and other “smaller” cell populations will become increasing apparent. Thus, future research should focus on (i) identifying molecular cues guiding lymphangiogenesis and neurovascular patterning within valvular tissue, (ii) developing model systems that faithfully recapitulate these multicellular and mechanical complexities, and (iii) determining how the neural-lymphatic interact with valvular endothelial and interstitial cells act during disease progression and remodeling.

Finally, the field owes much to pioneering efforts of Adrian Chester who emphasized the importance of these overlooked populations. Continued exploration of VECs, neural elements, and other minor cell types will not only deepen our mechanistic understanding of valve biology but also open avenues for diagnostics, therapeutics, and regenerative strategies tailored to the unique biology of the valve.

## Glossary

AoV: Aortic Valve
AS: Aortic Valve Stenosis
*α*SMA: Alpha Smooth Muscle Actin
AVR: Aortic Valve Replacement
BAV: Bicuspid Aortic Valve
BMP2: Bone Morphogenetic Protein 2
Cad-11: Cadherin-11
CAVD: Calcific Aortic Valve Disease
CCL21: C-C Motif Chemokine Ligand 21
CAD: Computer Aided Design
CD: Cluster of Differentiation
CWV: Cog-Wheel Valves
Dll4: Delta-like Canonical Notch Ligand 4
DMVD: Degenerative Mitral Valve Disease
ECM: Extracellular Matrix
EndMT: Endothelial-to-Mesenchymal Transition
eNOS: Endothelial Nitric Oxide Synthase
ERK: Extracellular Signal-Regulated Kinase
F4/80: Pan Macrophage Marker
FOXC2: Forkhead Box Protein 2
GAP: GTPase Activating Protein
GEF: Guanine Nucleotide Exchange Factor
GTP: Guanine Triphosphate
ICAM-1: Intercellular Adhesion Molecule 1
Jag1: Jagged1
KLF2: Krüppel-like Factor 2
KLF4: Krüppel-like Factor 4
LEC: Lymphatic Endothelial Cells
LYVE1: Lymphatic Vessel Endothelial Hyaluronan Receptor-1
MGP: Matrix Gla Protein
MHC: Major Histocompatibility Complex
MR: Mitral Valve Regurgitation
MV: Mitral Valve
NO: Nitric Oxide
NOTCH1: Neurogenic locus notch homolog protein 1
PDGF-*β*: Platelet Derived Growth Factor Beta
PDPN: Transmembrase *O*-glycoprotein Podoplanin
PFKFB3: 6-Phosphofructo-2-kinase/fructose-2,6-biphosphatase 3
PI3-Kinase: Phosphoinositide 3-kinase
PROX1: Prospero homeobox-1
Rho-Kinase (or) ROCK: Rho-associated Protein Kinase
RUNX2: Runt-related Transcription Factor 2
RBPJ: Recombination Signal Binding Protein for Immunoglobulin Kappa J Region
SMAD1: SMAD Family Member 1
SMC: Smooth Muscle Cells
Sox9: SRY-Box Transcription Factor 9
TEHV: Tissue Engineered Heart Valves
TGF-*β*: Transforming Growth Factor Beta
TKs: Tachykinins
VCAM-1: Vascular Cell Adhesion Molecule 1
VEC: Valvular Endothelial Cells
VEGFR 3: Vascular Endothelial Growth Factor Receptor 3
VIC: Valvular Interstitial Cells
VIP: Vasoactive Intestinal Polypeptide
Wnt: Wingless-related integration site.
